# A Case of Neuritis Simulating Progressive Muscular Atrophy Associated with Nystagmus and Increased Knee Jerks

**Published:** 1894-11

**Authors:** Harold N. Moyer

**Affiliations:** Chicago, Ill.


A CASE OF NEURITIS SIMULATING PROGRESSIVE MUSCULAR ATROPHY ASSOCIATED WITH NYSTAGMUS AND INCREASED KNEE JERKS. By Harold N. Moyer, M. D. Chicago, Ill.—The following singular grouping of symptoms came under our observation a short time since, and we deem it worthy of report because we have not seen such described in the literature nor has a similar case ever come under our observation:

A young woman 21 years of age presented herself at the clinic for nervous diseases of Rush Medical College, complaining of a progressive loss of strength and wasting of the hands and arms. She stated that the trouble had come on four years before. There had been no injury or serious sickness. At the time her occupation, that of a servant, required her to have her hands frequently in cold water. At first there was much pain in the hands, which would dart up the forearm; associated with this condition was numbness and tingling. After a time she noticed a progressive loss of strength, and still later that the hands and forearms were diminishing in size. As the disease advanced the pain and numbness ceased, giving place to a marked loss of sensation.

At the present time the hands show a marked loss of muscular structure; the thenar and hypothenar eminences have for the most part disappeared. The spaces between the metacarpal bones are deeply hollowed. The hands are commonly held in a semi-flexed position (claw-like) but there is no contraction of the flexor tendons. A little motion is possible with the hands and the patient has a feeble grasp. The strength of flexors is practically *nil.* The forearms are much wasted and the extent of atrophy is exactly the same in both arms. Fibrillary tremors are occasionally to be seen. The arm muscles have escaped; the biceps, triceps and muscles of the shoulder girdle are all intact. The supinator and pronator are not involved. Sensation in the left arm is abolished as high as the middle third. Tactile, common, pressure and temperature senses are equally involved, though deep pressure seems to be preserved to some extent and the muscular sense is not markedly impaired. The right arm is similarly involved, the anesthesia reaching to the shoulder and involving a portion of the surface of the chest at and below the axilla, corresponding to the distribution of the nerve of Wrisberg and the lesser internal cutaneous. The electrical reactions are altered in different degrees; in some muscles there is complete reaction of degeneration (interossei and muscles of the little finger and thumb). In some of the flexor muscles there is only a partial reaction of degeneration though in all affected muscles a. c. c. precedes c. c. c. Faradic irritability is mostly decreased or even absent (interossei) but in a few muscles seems slightly increased. Station is good, sensibility is elsewhere intact. The general health is good and the functions of circulation, respiration and digestion are not affected. Nystagmus is present, vision is good and the eye-grounds are normal in both eyes. The patellar tendon reflex is markedly exaggerated in both legs, and prolonged ankle clonus is present in both feet.

A brief glance at the foregoing reveals a very anomalous symptom grouping. That the trouble with the forearms and hands was a peripheral neuritis is, we think, shown by the fact that there was at first pain and paresthesia succeeded by a loss of sensation which still persists, and that the anesthesia is approximately coincident anatomically with the muscular atrophy. On the other hand, the distribution of the paralysis is exactly such as we get in acute polio-myelitis, of the lower arm type of Remak, in which the supinators and pronators escape, while the disease is perfectly symmetrical on the two sides. This is hardly to be looked for in neuritis, though it does sometimes occur; indeed, it is uncommon in polio-myelitis. Again, the gradual onset speaks in favor of a neuritis. While the polio-myelitis may have a gradual progress, it is usually by successive steps; as each group of cells are successively attacked there is a rapid disappearance of corresponding muscles. In this case there seems to have been an even progress in the wasting similar to that noted in the essential forms of progressive muscular atrophy. In this connection we might premise that in some cases it is practically impossible to make a diagnosis between peripheral disease and anterior horn lesion.

The nystagmus and exaggerated reflexes probably have no connection with the peripheral neuritis. Unfortunately, the history of the patient does not throw any light on the possible origin of these symptoms. Had the patient presented herself with these signs alone, we should have felt justified in making a diagnosis of disseminated sclerosis, and such in fact we believe the case to be. Indeed, there is nothing to be said against the view that this may be an instance of *sclerose en plaques*, in which the peripheral nerves share as well as the central nervous system. There is just one thing that may be said against this diagnosis, and that is the symmetrical character of the disease which in disseminated sclerosis is decidedly exceptional.—*Journal of American Medical Association.*

